# Nosological delineation of congenital ocular motor apraxia type Cogan: an observational study

**DOI:** 10.1186/s13023-016-0486-z

**Published:** 2016-07-29

**Authors:** Sarah Wente, Simone Schröder, Johannes Buckard, Hans-Martin Büttel, Florian von Deimling, Wilfried Diener, Martin Häussler, Susanne Hübschle, Silvia Kinder, Gerhard Kurlemann, Christoph Kretzschmar, Michael Lingen, Wiebke Maroske, Dirk Mundt, Iciar Sánchez-Albisua, Jürgen Seeger, Sandra P. Toelle, Eugen Boltshauser, Knut Brockmann

**Affiliations:** 1 Interdisciplinary Pediatric Center for Children with Developmental Disabilities and Severe Chronic Disorders, Department of Pediatrics and Adolescent Medicine, University Medical Center, Robert Koch Str. 40, 37075 Göttingen, Germany; 2 Sozialpädiatrisches Zentrum, Evangelisches Krankenhaus, Düsseldorf, Germany; 3 Sozialpädiatrisches Zentrum, SLK-Kliniken, Heilbronn, Germany; 4 Sozialpädiatrisches Zentrum, Coburg, Germany; 5 Pediatric Practice, Offenburg, Germany; 6 Sozialpädiatrisches Zentrum, University Medical Center, Würzburg, Germany; 7 Pediatric Practice, Mühlacker, Germany; 8 Sozialpädiatrisches Zentrum, University Medical Center, Dresden, Germany; 9 Department of Pediatric Neurology, University Children’s Hospital, Münster, Germany; 10 Sozialpädiatrisches Zentrum, Städtisches Klinikum Dresden-Neustadt, Dresden, Germany; 11 Department of Pediatrics and Adolescent Medicine, Euregio-Klinik Grafschaft Bentheim Holding GmbH, Nordhorn, Germany; 12 Sozialpädiatrisches Zentrum, St. Marien-Hospital, Düren, Germany; 13 Department of Pediatric Neurology, University Children’s Hospital, Tübingen, Germany; 14 Sozialpädiatrisches Zentrum Frankfurt Mitte, Frankfurt am Main, Germany; 15 Department of Pediatric Neurology, University Children’s Hospital, Zurich, Switzerland

**Keywords:** Congenital ocular motor apraxia, Molar tooth sign, Joubert syndrome

## Abstract

**Background:**

The nosological assignment of congenital ocular motor apraxia type Cogan (COMA) is still controversial. While regarded as a distinct entity by some authorities including the Online Mendelian Inheritance in Man catalog of genetic disorders, others consider COMA merely a clinical symptom.

**Methods:**

We performed a retrospective multicenter data collection study with re-evaluation of clinical and neuroimaging data of 21 previously unreported patients (8 female, 13 male, ages ranging from 2 to 24 years) diagnosed as having COMA.

**Results:**

Ocular motor apraxia (OMA) was recognized during the first year of life and confined to horizontal pursuit in all patients. OMA attenuated over the years in most cases, regressed completely in two siblings, and persisted unimproved in one individual. Accompanying clinical features included early onset ataxia in most patients and cognitive impairment with learning disability (*n* = 6) or intellectual disability (*n* = 4). Re-evaluation of MRI data sets revealed a hitherto unrecognized molar tooth sign diagnostic for Joubert syndrome in 11 patients, neuroimaging features of Poretti-Boltshauser syndrome in one case and cerebral malformation suspicious of a tubulinopathy in another subject. In the remainder, MRI showed vermian hypo-/dysplasia in 4 and no abnormalities in another 4 patients. There was a strong trend to more severe cognitive impairment in patients with Joubert syndrome compared to those with inconclusive MRI, but otherwise no significant difference in clinical phenotypes between these two groups.

**Conclusions:**

Systematical renewed analysis of neuroimaging data resulted in a diagnostic reappraisal in the majority of patients with early-onset OMA in the cohort reported here. This finding poses a further challenge to the notion of COMA constituting a separate entity and underlines the need for an expert assessment of neuroimaging in children with COMA, especially if they show cognitive impairment.

**Electronic supplementary material:**

The online version of this article (doi:10.1186/s13023-016-0486-z) contains supplementary material, which is available to authorized users.

## Background

The term congenital ocular motor apraxia (COMA) was introduced by Cogan in 1952 when he described a particular disorder of voluntary horizontal gaze in four children [1]. “Inability to turn the eyes voluntarily in a direction for which there is full involuntary … control” together with compensatory, jerky head movements constituted the clinical hallmark of this condition. COMA is related to the inability to initiate saccades.

During subsequent decades a wide range of disorders associated with ocular motor apraxia (OMA) was recognized [2]. However, several reviews and case reports of congenital OMA, alternatively called “infantile-onset saccade initiation delay”, stressed frequent consistent co-occurrence with early-onset cerebellar ataxia and global developmental delay [3–9]. Typically, OMA and ataxia resolve over the years, while cognitive impairment persists to variable degree [4, 7–9]. Thus, while OMA constitutes a symptom, not a diagnosis [2], a concept of COMA emerged indicating that this condition frames a clinical entity and a genetic disorder with autosomal recessive inheritance. The Online Mendelian Inheritance in Man (OMIM) catalog of human genes and genetic disorders provides a separate coding number (%257550) for COMA. However, no gene associated with isolated COMA has been identified yet.

On the other hand, early-onset OMA is a frequent symptom of Joubert syndrome (JBTS), accompanied by infantile muscular hypotonia, early-onset ataxia, neonatal breathing abnormalities with episodic tachypnea and apnea, as well as developmental delay/intellectual disability [10–12]. Thus there is considerable phenotypic overlap of COMA and JBTS. Yet, both conditions are separated on the basis of neuroimaging characteristics.

MRI in COMA was reported to be either normal or show a vermian hypoplasia, preferentially of the inferior portion of the vermis [13, 14]. A recent review of studies performed between 1952 and 2012 included 91 patients with a clinical phenotype consistent with COMA and found MRI to be allegedly normal in 55 of them [15].

In contrast, a highly characteristic neuroimaging feature of JBTS in axial MRI of the brainstem was recognized in 1997 and designated the “molar tooth sign” (MTS) [16]. The “molar tooth” derives from the combination of elongated, thickened and horizontalised superior cerebellar peduncles; hypo-/dysplasia of the cerebellar vermis; and an abnormally deep interpeduncular fossa at the section of the brainstem isthmus and upper pons [12]. It is now considered to be pathognomonic for JBTS [17].

Our study presented here originally aimed at identification of the gene associated with COMA. However, re-evaluation of clinical and neuroimaging data of patients recruited with the diagnosis of COMA indicated that delineation of COMA versus JBTS and clarification of the nosological relationship between these two conditions are mandatory and a prerequisite for genetic investigations in COMA.

## Methods

This study was approved by the ethics committee of the Faculty of Medicine, University of Göttingen (file no. 19/5/14). Written informed consent was obtained from all families.

### Patient cohort

Patients diagnosed as having COMA were collected (i) from the cohort of patients of the Department of Pediatric Neurology, University Medical Center Göttingen (*n* = 4) and (ii) using an email based acquisition of rare neurological disorders in childhood (“Erhebung Seltener Neurologischer Erkrankungen im Kindesalter, ESNEK”) [18] from pediatric neurologists in Germany (*n* = 16) and Switzerland (*n* = 1).

Inclusion criteria for the definite COMA cohort comprised written informed consent of the parents or the patient or both, early-onset OMA (diagnostic recognition within the first year of life), and availability of an MRI in technical quality adequate for assessment of especially brainstem and cerebellum. We did not include patients with the MTS already recognized on MRI, thus with a preexisting diagnosis of JBTS.

### Clinical and qualitative neuroimaging analysis

Demographic data, neurological features and information about the developmental stage were compiled by review of the clinical histories and by clinical-neurological follow-up investigations. Information about ophthalmological features was collected from neuro-ophthalmological or pediatric-neurological reports. Cognitive function was assessed using standardized neuropsychological tests whenever possible. Otherwise, the stage of mental developmental was appraised from the patient’s history, clinical examination, and reports from nursery or school. Additional data were collected in an interview with the parents conducted by telephone using a standardized questionnaire (Additional file 1).

A comparison concerning qualitative neurological features and quantifiable developmental data of the patient subgroups with MTS on MRI vs. inconclusive MRI was performed using T-test for independent variables and Fisher´s exact test.

All MRI data sets were analyzed independently by two pediatric neurologists with experience in neuroimaging of the brainstem and cerebellum (EB, KB) [19]. All available imaging sequences in axial, coronal and sagittal orientation were scrutinized with focus on size and position of the superior cerebellar peduncles, hypo-/dysplasia of the cerebellar vermis, cerebellar cysts, brainstem morphology including shape of the interpeduncular fossa at the section of the brainstem isthmus and upper pons, size and shape of the 4th ventricle, and any other cerebellar or cerebral malformations.

## Results

A total of 28 patients were recruited via email based acquisition and from our own department. Among these, critical reappraisal of disease course and clinical features revealed that phenotypes were not consistent with COMA in three cases (e.g., onset of OMA later than 2^nd^ birthday, neuroophthalmological features not consistent with OMA). In additional three patients cranial MRI was not available in sufficient technical quality. One patient already had a molecular genetic diagnosis of JBTS. Thus 21 patients were included in this study (13 male, 8 female, two pairs of siblings, mean age 10 years 3 months, age range 2 years 8 months - 24 years). Parental consanguinity was reported in one patient (#7). Table 1 summarizes clinical and neuroimaging features of all 21 patients. The period during which COMA was diagnosed in these patients spans from 1992 to 2013.

**Table 1:** Clinical and neuroradiological features of 21 patients with "congenital ocular motor apraxia type Cogan" (COMA)

Patient # (origin)	Sex	Current age (years)	Affected/unaffected siblings	Development		Neurological findings				MRI features	Diagnostic assignment
				Unaided walking at age (months)	Speech delay	Ocular apraxia [age at onset (months)/course/age at disappearance]	Early onset ataxia	Cognitive development	Seizures		
1 (A)	f	4	0/1	18	Yes	3/↓/-	Yes	Learning disability	No	MTS, vermian hypo-/dysplasia	JBTS
2 (D)	m	7	0/0	27	Yes	8/↓/-	Yes	Learning disability	No	MTS, superior vermian hypoplasia, slightly enlarged external csf spaces	JBTS
3 (D)	f	12	0 / 1	20	No	3/↓/-	No	Normal	No	Normal	COMA
4 (D)	m	18	0/1	30	Yes	3/↓/-	Yes	Learning disability	No	MTS, superior vermian dysplasia	JBTS
5 (TR)	f	5	0/2	30	Yes	4/↓/-	Yes	Intellectual disability	No	MTS, superior vermian hypo-/dysplasia	JBTS
6 (D)	f	4	0/1	27	No	6/↓/-	Yes	Normal	Yes	vermian dysplasia, otherwise normal	COMA
7 (TR)	m	18	0/1	20	Yes	6/↔/-	Yes	Normal	No	Cerebellar cysts, cerebellar hypoplasia, square 4th ventricle	Poretti-Boltshauser syndrome
8 (D)	m	24	1 (#9)/1	24	Yes	6/↓/4 years	Yes	Intellectual disability	No	MTS, vermian hypo-/dysplasia	JBTS
9 (D)	f	21	1 (#8)/1	14	Yes	11/↓/5 years	Yes	Learning disability	No	MTS, otherwise normal	JBTS
10 (T)	m	9	0/3	24	Yes	6/↓/-	Yes	Learning disability	Yes	Inferior vermian dysplasia, large cerebellum, slight caudal extension of cerebellar tonsils	COMA
11 (D)	m	16	0/3^a^	27	Yes	2/↓/-	No	Normal	No	Normal	COMA
12 (D)	f	6	0/0	20	Yes	10/↓/-	Yes	Normal	No	Normal	COMA
13 (D/UK)	m	2	0/2^a^	27	No	8/↓/-	Yes	Normal	No	MTS, otherwise normal (mild superior vermian hypo-/dysplasia??)	JBTS
14 (CH)	m	7	0/1	24	Yes	6/↓/-	Yes	Normal	No	mild vermian dysplasia, otherwise normal	COMA
15 (D)	m	6	0/2	30	Yes	8/↓/-	Yes	Intellectual disability	No	MTS, superior vermian dysplasia	JBTS
16 (D)	m	22	1 (#17)/1	30	Yes	6/↓/-	Yes	Normal	Yes	MTS, vermian hypo-/dysplasia	JBTS
17 (D)	m	17	1 (#16)/1	42	Yes	3/↓/-	Yes	Low normal	No	MTS, superior vermian hypo-/dysplasia	JBTS
18 (D)	f	23	0/1	24	No	4/↓/-	Yes	Learning disability	No	Normal	COMA
19 (D)	f	6	0/1 + 1^a^	14	No	6/↓/-	No	Normal	No	Superior vermian dysplasia, otherwise normal	COMA
20 (R/K)	m	10	0/1 + 1^a^	48	Yes	5/↓/-	Yes	Intellectual disability	No	Enlarged ventricles, dysmorphic basal ganglia, hypoplastic corpus callosum, abnormal proportions of brain stem	Brain malformation suspicious of tubulinopathy
21 (D)	m	6	0/1	36	Yes	8/↓/-	Yes	Learning disability	No	MTS, callosal agenesis, vermian hypo-/dysplasia, hippocampal malrotation, dysplastic tectal plate	JBTS

*Abbreviations*: *A* Albanian origin, *CH* Swiss origin, *D* German origin, *K* Kazakh origin, *R* Russian origin, *T* Turkish origin, *UK* British origin, *m* male, *f* female, ↓ attenuating, ↔ unchanged, *MTS* molar tooth sign, *JBTS* Joubert syndrome, ^a^ half-siblings

### Ophthalmological features

In all patients OMA was recognized by attending physicians during the child´s first year of life. Most children (15 of 21 cases) presented with visual problems already in the first months of life, before OMA was definitely diagnosed, occasionally giving rise to the concern of severe visual impairment or even blindness. Initial visual symptoms included lack of fixation and visual pursuit (*n* = 12), nystagmus (*n* = 5), head tilting (*n* = 3), and strabism (*n* = 2), with ages at onset ranging from 4 weeks to 6 months.

Inability to perform normal voluntary saccades was confined to horizontal pursuit, vertical OMA was not observed in any of our patients. In almost all subjects OMA gradually ameliorated over the years, with complete cessation in two siblings (#8 and #9) at age 4 and 5 years. Additional oculomotor features comprised nystagmus in three patients and Duane syndrome in two siblings (#8 and #9).

### Neurological features and developmental course

In most patients, early-onset cerebellar ataxia together with muscular hypotonia constituted further clinical hallmarks with gradual amelioration during the first decade, while general clumsiness persisted. Motor development was delayed in most cases, unsupported walking was achieved at ages ranging from 14 months to four years (mean 25 months). All subjects showed gradual ongoing improvement of motor skills. Development of both, language perception and expressive speech was delayed as well in all but two cases. An impairment of cognitive development was observed in 11 cases comprising learning disability (IQ 70-84; *n* = 7) or intellectual disability (IQ <70; *n* = 4).

### Neuroimaging features

**Fig. 1:**
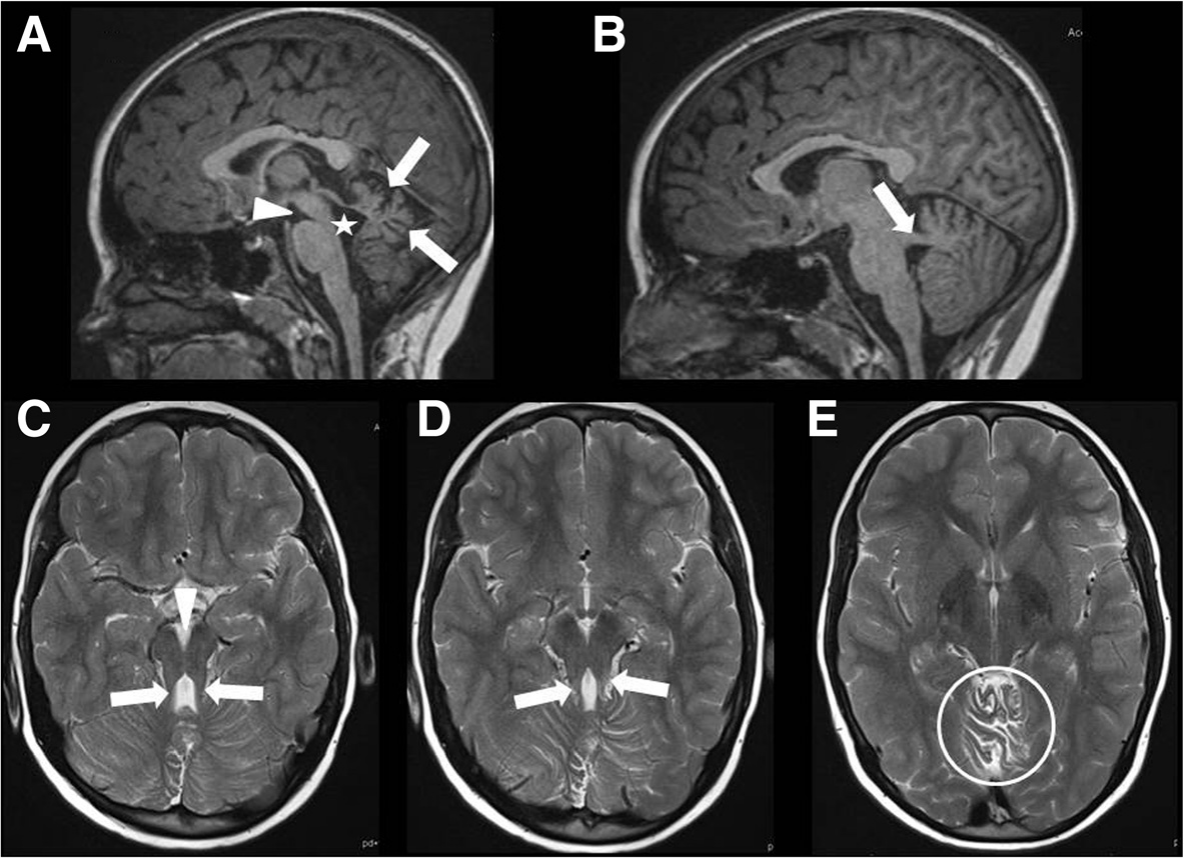
Molar tooth sign on MRI of an infant with Joubert syndrome. **a**, **b** Sagittal T1-weighted and **c**-**d** axial T2-weighted MRI of patient #1 at age 8 months. **a** Midsagittal slice shows vermian hypo-/dysplasia (arrows), rostral shifting of the fastigium (star), and deep interpeduncular fossa (arrowhead). **b** Parasagittal slice displays thickened and horizontalized superior cerebellar peduncles (SCP)(arrow). **c**-**e** Axial slices show deep interpeduncular fossa (arrowhead) and elongated SCP (arrows), resulting in “molar tooth sign”, and irregular folia of upper vermis (circle). These MRI features indicate Joubert syndrome

**Fig. 2:**
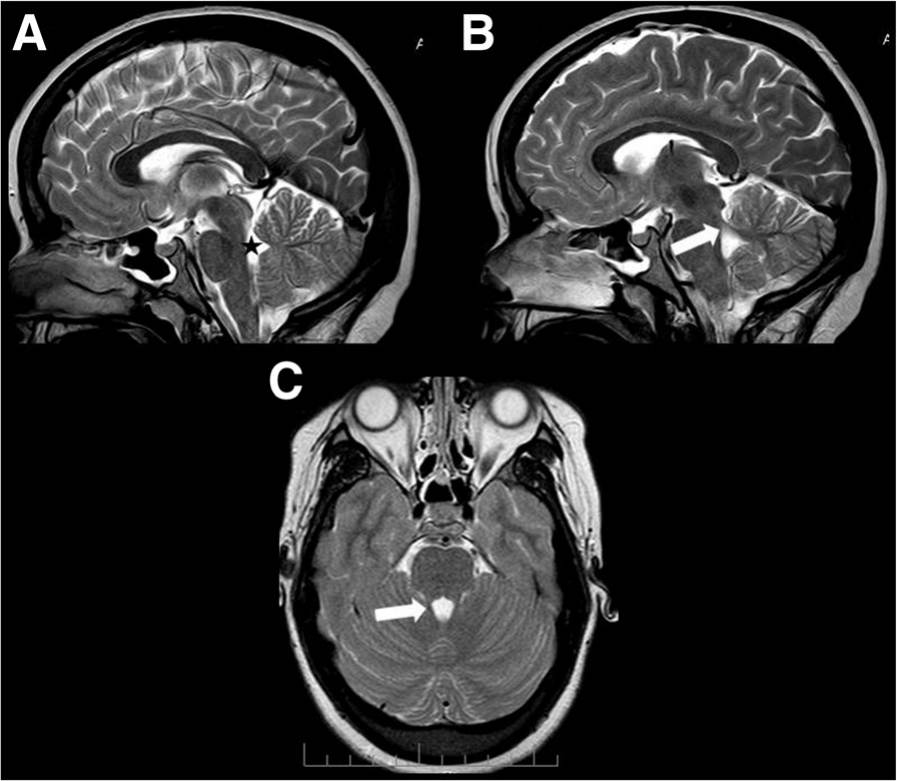
Normal MRI in a 19-year-old patient with COMA. **a**, **b** Sagittal and (**c**) axial T2-weighted MRI of patient #18 at age 19 years. **a** Midsagittal slice shows normal position of fastigium (star) and well developed vermis. **b** Parasagittal slice displays thin superior cerebellar peduncles (SCP) in oblique position (arrow). **c** Axial slice shows normal SCP (arrow). This MRI is normal

Systematic analysis of all MRI data sets revealed a MTS in 11 patients, indicating JBTS (Fig. 1). Among these patients with MTS, 10 additionally had vermian hypo-/dysplasia, predominantly of the superior part of the vermis cerebelli, and one of these cases showed agenesis of the corpus callosum. In one patient with MTS, MRI was otherwise normal. In one male patient (#7) cerebellar dysplasia, cerebellar cysts, and enlargement as well as square shape of the 4^th^ ventricle clearly pointed to Poretti-Boltshauser syndrome [20, 21]. One patient had dysmorphic basal ganglia, abnormal shape of frontal horns, abnormal corpus callosum, reduced pontine prominence, and enlarged ventricles, suspicious of a tubulinopathy [22]. In 4 cases MRI revealed vermian hypo-/dysplasia as sole abnormality. MRI was normal in the remaining 4 patients (Fig. 2).

### Comparison of clinical phenotypes of COMA vs. JBTS

A comparison of qualitative clinical features and quantitative developmental data revealed no difference between the subgroup of patients with inconclusive MRI vs. those with MTS on MRI concerning present age, age at diagnosis of COMA, and amelioration of OMA (Table 2). Unsupported walking was attained at slightly higher ages in children with JBTS, and there was a strong trend to more severe cognitive impairment in JBTS. However, these differences did not reach statistical significance, possibly due to small case numbers.

**Table 2:** Comparison of clinical features in patients with Joubert syndrome vs. those with inconclusive MRI findings

Neuroradiological diagnosis	Joubert syndrome	Inconclusive MRI	T	p
Number of patients	11	8		
Sex	8 male, 3 female	3 male, 5 female		
Present age (years) [mean (SD)]	12.0 (8.3)	10.4 (6.4)	0.46	0.65
Age at diagnosis of COMA (months) [mean (SD)]	6.2 (2.7)	5.4 (2.4)	0.67	0.51
Amelioration of OMA	100 % (11/11)	100 % (8/8)		
Unsupported walking at age (months) [mean (SD)]	28.0 (7.7)	22.5 (4.3)	1.82	0.09
Cognitive development:			Fisher´s exact test: *p* = 0.055	
Normal	3	6		
Learning disability or intellectual disability	8	2		

*Abbreviations*: *COMA* congenital ocular motor apraxia, *OMA* ocular motor apraxia, *SD* standard deviation, *T* T value from T-test for independent variables

### Various features

A 17-year-old boy with MTS (# 4) had retinal coloboma, neonatal breathing dysregulation, and persistently elevated liver enzymes. These features are well-known in JBTS with liver involvement due to *TMEM67* mutations [23], but had not been recognized earlier as pointing to JBTS in our patient. Patient # 15 with MTS had congenital clubfoot, which was occasionally reported in JBTS, namely in a 4-year-old girl with a homozygous frameshift mutation in the *RPGRIP1L* gene [24]. No other clinical or laboratory features of JBTS, including polydactyly, facial dysmorphism, neonatal breathing dysregulation, skeletal dysplasia, cystic dysplastic kidneys, liver fibrosis, midline oral or facial defects, and oral soft tumors were observed in any of the remaining 9 patients with MTS.

## Discussion

In our cohort of 21 patients with clinical phenotypes consistent with COMA a reappraisal of MR images primarily judged to be normal revealed a MTS in 11 patients. Thus the majority of patients clinically diagnosed to have COMA can in fact be assigned to JBTS. All these MR images had previously been analyzed by board-certified radiologists or neuroradiologists, in some cases with explicit question whether there is a MTS, and had been rated as normal.

Several factors contribute to the difficulties in recognition of the MTS in our cohort: The molar tooth is a characteristic, but subtle MRI sign which is easily missed. It was recognized as a typical hallmark of JBTS in neuroimaging only in 1997, 28 years after first description of JBTS as a clinical syndrome [16]. MRI was introduced in clinical neurology in the mid-1980s. According to a medline search seven articles dealing with JBTS and MRI were published in scientific journals from 1989 until 1997 without perceiving the MTS. In two siblings (#8 and #9) of our cohort the first MRI was performed before 1997. Finally, technical shortcomings of the MRI investigation including inadequate angulation or slice thickness may additionally hamper recognition of the MTS.

In two additional patients of our cohort re-evaluation of MRI revealed otherwise specific neuroimaging features. An 18-year-old man (#7) with clinical phenotype consistent with COMA had clear MRI characteristics of Poretti-Boltshauser syndrome, and a 10-year-old boy showed neuroimaging features pointing to a tubulinopathy. Appropriate molecular genetic investigations are under way in these two cases. In the remaining 8 patients with clinical phenotype consistent with COMA MRI revealed no conclusive findings.

These results strongly underline that a brain MRI of a child with COMA needs to be reviewed by a neuroradiologist or neurologist with experience in pediatric posterior fossa diseases, particularly malformations. As has been shown for brain MRI in children with cerebral palsy the accuracy of neuroimaging reports clearly depends on the expertise of those reporting [25]. An expert review is even more important in a child with COMA and abnormal cognitive function, as the chance of a MTS is much higher in this subgroup of children with COMA (73 % vs. 25 % in our cohort).

Among the four patients originally described by Cogan in 1952, no other neurologic symptoms were present in two, while one had convulsions shortly after birth without neurologic sequelae and one had a persistent extrapyramidal disorder assigned to intrauterine carbon monoxide poisoning [1]. The follow-up report of these 4 patients 14 years later stated difficulties in reading (due to OMA) and normal intelligence in three patients, with additional mild balance problems in one of them. The patient with carbon monoxide poisoning had spasticity, ataxia and borderline intellectual disability [26]. It is obvious from these clinical depictions that in his original report Cogan did not delineate a homogeneous clinical entity but rather described a clinical sign observed in a small heterogeneous group of patients. Once OMA was recognized by neurologists and ophthalmologists the relatively frequent co-occurrence of infantile-onset OMA (COMA) in sporadic and familial cases with early-onset ataxia and delay of psychomotor development resulted in the perception of COMA as an entity, namely a complex neurodevelopmental disorder with autosomal-recessive inheritance.

It has been doubted “whether Cogan-type oculomotor apraxia can exist as an isolated entity” [27], and a current concept assigns COMA to three main clinical conditions [28]: “a. In the “benign” or “idiopathic” variety of congenital ocular motor apraxia, neuroimaging is normal and there is no readily identifiable explanation for the disorder. Although the neurologic examination and intellect are usually normal, occasionally associated neurologic defects include hypotonia, motor and speech delay, and ataxia…b. Some patients with congenital ocular motor apraxia have a nonprogressive, noninherited structural abnormality of the brain, caused either by a developmental anomaly or by prenatal or perinatal insult. These include: dysgenesis of the cerebellar vermis or corpus callosum, inferior vermian hypoplasia, Dandy–Walker malformation, gray matter heterotopias, and perinatal ischemia. c. A variety of genetic disorders with multisystem involvement may present in infancy with congenital ocular motor apraxia. These include Joubert syndrome, Jeune syndrome (nephronophthisis, asphyxiating thoracic dystrophy, retinal degeneration, and ataxia), and a subset of patients with Leber congenital amaurosis, a retinal dystrophy.” [28] Poretti-Boltshauser syndrome and tubulinopathies are to be added to this list of genetic disorders. JBTS, Jeune syndrome, and Leber congenital amaurosis are all ciliopathies and most likely overlapping phenotypes of the same group of diseases.

In contrast to this spectrum of conditions related to COMA, acquired OMA especially occurs in a range of different genetic disorders comprising ataxia teleangiectasia (AT), ataxia with ocular motor apraxia 1 (AOA1), and ataxia with ocular motor apraxia 2 (AOA2). The associated genes *ATM* (AT), aprataxin (AOA1) and senataxin (AOA2) are involved in mechanisms of DNA repair.

## Conclusions

Our results indicate that a large subgroup of patients diagnosed with “benign” or “idiopathic” COMA in fact have JBTS, as the MTS was missed on MRI – which may easily happen. Therefore meticulous analysis of the MRI in patients with COMA is advisable. Furthermore we found no consistent differences in clinical phenotypes (developmental delay, intellectual impairment, ataxia) between patients with the MTS and those with “benign” or “idiopathic” COMA, apart from one case with Poretti-Boltshauser syndrome and one patient with clinical features pointing to JBTS which were however not previously recognized in their diagnostic value in these patients.

In the future, delineation of new clinical and genetic entities associated with early-onset OMA will possibly allow for assignment of hitherto ill-defined cases of “idiopathic” COMA to a specific diagnosis. Additional genetic studies using whole exome sequencing are needed to clarify whether a subset of cases with early-onset OMA without the MTS on MRI frames a nosological entity with a distinct genetic basis and autosomal recessive inheritance, possibly constituting a further part of the clinical and genetic spectrum of ciliopathies.

## Abbreviations

AOA1, ataxia with ocular motor apraxia 1; AOA2, ataxia with ocular motor apraxia 2; AT, ataxia teleangiectasia (AT); COMA, congenital ocular motor apraxia; JBTS, Joubert syndrome; MTS, molar tooth sign; OMA, ocular motor apraxia

## Additional file

Additional file 1: Congenital Ocular Motor Apraxia (COMA) – Questionnaire. (DOCX 26 kb)
