# Supplementary material for: Nosological delineation of congenital ocular motor apraxia type Cogan: an observational study
Source: Orphanet J Rare Dis. 2016 Jul 29;11:104. doi: 10.1186/s13023-016-0486-z (PMC4966602; doi:10.1186/s13023-016-0486-z)
Supplement: Additional file 1: — Congenital Ocular Motor Apraxia (COMA) – Questionnaire. (DOCX 26 kb) [file 13023_2016_486_MOESM1_ESM.docx]

| Reporting physician: | _____________________________________________ |
| --- | --- |
| *Patient data*  Date of birth: | Patient #:……..  month /year _____ / __________ |
| Sex: | □ female  □ male |
| *Birth*  Gestational age: | □ term  □ preterm (…. weeks GA) |
| Perinatal complications: | □ no  □ yes  If any, which? _______________________________ |
| *Family history*  Other family members affected: | □ no  □ yes  If any, who? _________________________________  _____________________________________________ |
| Consanguinity of parents: | □ no  □ yes |
| *Developmental data*  Age at unaided walking: | _____ years _____ months |
| Speech delay: | □ no  □ yes |
| *Ocular findings* |  |
| Ocular motor apraxia: | Onset at age _____ months |
| Course: | □ attenuating  □ normal  □ increasing |
| Jerking head movements: | □ no  □ yes, at age …. |
| Nystagmus: | □ no  □ yes, at age…. |
| Involvement of vertical eye movements: | □ no  □ yes, at age…. |
| *Neurological findings*  Ataxia: | □ no  □ yes, □ trunc, □ limbs |
| Muscular hypotonia: | □ no  □ yes |
| Cognitive development: | □ normal  □ impaired  In case psychological test results available:  □ intellectual disability (IQ<70)  □ learning disability (IQ<85)  □ normal ((IQ>85) |
|  |  |
| Epilepsy: | □ no  □ yes |
| *Organ involvement*  Hepatic involvement: | □ no  □ yes |
| Elevated liver enzymes | □ no  □ yes  ALT: ___________ U/l  AST: ___________ U/l |
| Renal involvement: | □ no  □ yes |
| Elevated serum creatinine: | □ no  □ yes  creatinine: ______ mg/dl or ______ µmol/l |
| Polyuria/Polydipsia: | □ no  □ yes  □ unknown |
| *Other clinical findings*  Irregular breathing pattern in neonatal age (i.e. apnoe, tachypnoe): | □ no  □ yes  □ unknown |
| Retinal anomaly (i.e. chorioidoretinal coloboma?) | □ no  □ yes  □ unknown |
| Dysmorphic facial features? | □ no  □ yes  If any, which? _______________________________  _____________________________________________ |
| Skeletal features (i.e. polydactyly)? | □ no  □ yes  If any, which? _______________________________  _____________________________________________ |
| Other clinical symptoms/abnormalities? | □ no  □ yes  If any, which? _______________________________  _____________________________________________  _____________________________________________ |
| Previous genetic testing: | □ none  □ these tests were performed:  _____________________________________________  _____________________________________________  _____________________________________________  _____________________________________________  _____________________________________________ |
